# The association between continuous ambulatory heart rate, heart rate variability, and 24-h rhythms of heart rate with familial longevity and aging

**DOI:** 10.18632/aging.204219

**Published:** 2022-08-16

**Authors:** Janneke M. Wiersema, Annelies E.P. Kamphuis, Jos H.T. Rohling, Laura Kervezee, Abimbola A. Akintola, Steffy W. Jansen, P. Eline Slagboom, Diana van Heemst, Evie van der Spoel

**Affiliations:** 1 Section Gerontology and Geriatrics, Department of Internal Medicine, Leiden University Medical Center, Leiden, The Netherlands; 2 Department of Cell and Chemical Biology, Leiden University Medical Center, Leiden, The Netherlands; 3 Department of Geriatric Medicine, Catharina Hospital, Eindhoven, The Netherlands; 4 Section Molecular Epidemiology, Department of Biomedical Data Sciences, Leiden University Medical Center, Leiden, The Netherlands

**Keywords:** longevity, aging, continuous ambulatory measurements, heart rate, heart rate variability

## Abstract

Aging is associated with changes in heart rate (HR), heart rate variability (HRV), and 24-h rhythms in HR. Longevity has been linked to lower resting HR, while a higher resting HR and a decreased HRV were linked to cardiovascular events and increased mortality risk. HR and HRV are often investigated during a short electrocardiogram (ECG) measurement at a hospital. In this study, we aim to investigate the relationship between HR parameters with familial longevity and chronological age derived from continuous ambulatory ECG measurements collected over a period of 24 to 90 hours. We included 73 middle-aged participants (mean (SD) age: 67.0 (6.16) years), comprising 37 offspring of long-lived families, 36 of their partners, and 35 young participants (22.8 (3.96) years). We found no association with familial longevity, but middle-aged participants had lower 24-h HR (average and maximum HR, not minimum HR), lower amplitudes, and earlier trough and peak times than young participants. Associations in HR with chronological age could be caused by the aging process or by differences in environmental factors. Interestingly, middle-aged participants had a less optimal HRV during long-term recordings in both the sleep and awake periods, which might indicate that their heart is less adaptable than that of young participants. This could be a first indication of deteriorated cardiovascular health in middle-aged individuals.

## INTRODUCTION

Already in 1997, an inverse linear relationship between resting heart rate (HR) and life expectancy among different mammalian species was observed by Levine [1, 2]. The inverse association between HR and life expectancy has been attributed to metabolic rate, which is generally higher in smaller mammals and lower in larger mammals and directly correlated with HR [1, 2]. Fueled by these observations, subsequent studies in humans investigated correlations between resting HR and lifespan among individuals [3]. A higher resting HR was found to be associated with cardiovascular events and increased mortality risk, while a lower resting HR has been associated with health and longevity [4–10]. Several factors regulate HR, and it is likely that these may independently affect health and longevity. However, a lower HR may also beneficially affect the cardiovascular system directly, amongst others by decreasing ventricular load, oxygen consumption, aortic/arterial stiffness, and endothelial stress. With chronological age, the maximal HR decreases while resting HR was found to be relatively stable with age in healthy individuals [3, 11].

Aging is an important risk factor for cardiovascular disease (CVD), one of the leading causes of death worldwide [12]. One mechanism that has been proposed to contribute to the increased prevalence of CVD in the aging population is autonomic imbalance [13]. Functioning of the autonomic nervous system (ANS) can be assessed by various measurements of heart rate variability (HRV), which are indicative of the balance between sympathetic and parasympathetic nervous system activity [14]. Chronological aging has been linked to a progressive decline in HRV [15–18] and a decreased HRV is associated with a number of adverse cardiovascular outcomes such as heart failure, hypertension, and sudden cardiac death [19–24].

Aging also influences circadian rhythms in the cardiovascular system [19, 25–27]. In healthy humans, HR shows a clear circadian pattern with peaks in the morning and in the early evening [25]. Changes associated with aging include a reduced amplitude and an earlier phase of the circadian rhythm in HR, which are thought to rely on age-dependent disruptions of ANS functioning [19, 25, 26, 28]. Daily variations in the cardiovascular system are thought to underlie the increased risk of cardiovascular events in the morning, such as coronary heart disease and myocardial infarcts [19, 25, 26, 29, 30].

Familial longevity is associated with reduced prevalence and incidence of CVD [31–33]. This was also found in the Leiden Longevity Study (LLS), which includes offspring of nonagenarian siblings and their current partners [34]. Interestingly, studying this full cohort showed that the offspring had a lower prevalence of multiple age-related diseases, including myocardial infarction and hypertension, compared to their partners [35]. It is however not known whether offspring and partners also differ in resting HR, in parameters of HRV or in daily 24-h rhythms in HR.

Parameters of HR and HRV are often investigated during a short electrocardiogram (ECG) measurement at the study center or in the hospital, but not continuously over a longer period while individuals continue with their daily lives. In addition, HRV is often measured using short-term (~5 min) time-domain metrics such as the SDNN [14]. However in the current study, we collected continuous ambulatory ECG measurements over a time period of 24 to 90 hours (h) in young and middle-aged participants from the Switchbox Leiden Study [36]. Parameters of HR, HRV, and 24-h HR rhythms were extracted from the data. We aim to investigate the association between these parameters with familial longevity by comparing healthy middle-aged offspring of long-lived parents with their current partners, who were recruited from the Leiden Longevity Study [34, 35]. Additionally, we compare this group of middle-aged participants with younger controls from the general population to investigate the associations between parameters of HR, HRV, and 24-h HR rhythms with chronological age. We used detrended fluctuation analysis (DFA) as a measure for HRV, which is a non-linear measurement to quantify the unpredictability of a time-series and novel for this type of study [14, 37, 38].

## METHODS

### Study participants

Continuous ambulatory electrocardiogram (ECG) measurements were collected in the Switchbox Leiden Study [36]. In this study, we included 135 middle-aged (age range = 52–83 y) participants who were recruited from the Leiden Longevity Study (LLS). Of these 135 participants, 74 were offspring of long-lived families and 61 were the current partners of this offspring. The LLS is a family-based study consisting of 421 Caucasian families with at least two long-lived siblings (men ≥89 years and women ≥91 years) together with their offspring and the offspring’s partners without any selection on health or demographics [34]. The offspring of long-lived parents are likely genetically predisposed to become long-lived, while their partners are not. Moreover, the offspring and partners are likely to share similar environment, lifestyle, and age, which makes the partners an appropriate control group. By comparing offspring and partners, we can investigate the association between HR parameters and familial longevity. Exclusion criteria for the Switchbox Leiden Study were recent weight loss, recent trans-meridian flight, recently performed shift work, having a fasting plasma glucose above 7 mmol/L, having chronic renal, hepatic, or endocrine disease, or using medication known to influence lipolysis, thyroid function, glucose metabolism, GH or IGF-1 secretion, and/or any other hormonal axis, having anemia (hemoglobin <7.1 mmol/L), or having had a blood donation within the last two months.

In the Switchbox Leiden Study, we also included a control group of 48 young (age range = 18–40 y) individuals in order to investigate associations with chronological age by comparing them to the middle-aged LLS participants (offspring and partners combined). Most young individuals were students or employees of the Leiden University. Three study rounds with young participants were performed with variations in the protocol: in two of these study rounds, younger participants were excluded if they consumed more than 3 units of alcohol per day or based on self-reported cardiovascular disease. However, the study protocol for all young participants was the same as for the middle-aged participants concerning the ECG measurements.

The protocol of the Switchbox Leiden Study was approved by the Medical Ethical Committee of the Leiden University Medical Centre and performed according to the Helsinki declaration. All participants gave written informed consent for participation. Ultimately, after data cleaning, 37 middle-aged offspring, 36 middle-aged partners, and 35 young participants were included in the current analyses.

### Study protocol

From 18:00 h at the first study day onwards, participants wore an Equivital EQ02 lifemonitor (EQ02). This device continuously collected various physiological data including ECG over a period of up to almost four days (until 12:00 h on the fifth day of the study) while the participants were at home [39]. During the study period, participants recorded, amongst other, the timing of meals, exercise, going to bed, getting up, charging the Equivital EQ02 lifemonitor device in a paper diary.

A group of 38 middle-aged participants followed a slightly different protocol of which 15 participants (6 offspring and 9 partners) were included in the current analyses after data cleaning. These 15 participants with a deviated protocol came back to the research center on the second study day to participate in a serial blood sampling study during which blood samples were collected every 10 min during 24 h starting at 09:00 h [36]. After this hospitalization, they returned home and followed the remaining protocol the same way as the other participants. Therefore, for these 15 participants, only the data that was collected at home for 1.5 day (starting at 00:00 h at the fourth study day until 12:00 h on the fifth day) was included in the current analyses.

### Data cleaning

The EQ02 lifemonitor recorded continuously ECG, core body temperature, skin temperature, and accelerometry data [40]. The ECG data was recorded with a frequency of 256 Hz. SEM files containing raw ECG data from EQ02 were uploaded to the Vivosense modular physiological monitoring and analysis platform (Vivonoetics, San Diego, USA). Each SEM file was visually inspected via the cardiac layout. After visual inspection, all separate SEM files per participant were merged. In the complete merge, the gain of each channel was adjusted to represent physiological values. The software program Vivosense automatically detects R peaks, which are defined as the highest amplitude of the R wave in the QRS complex, in the raw ECG data. Next, automatic artefact marking (RR1) was used to clean the complete merge. The upper HR limit was set to 220, the lower to 30, ectopic beat detection was turned on, noise filtering was set to high and the maximal interpolation length (s) was set to zero. Date- and timestamped HR data, RR data, and periods automatically marked as artefacts, were exported from Vivosense as a Comma Separated Values (CSV) file. These files were imported into R version 3.6.2 [41] and the artefacts annotated in Vivosense were removed. The EQ02 device had to be charged two times per day for at least half an hour, so based on visual inspection and the information in the diary of the participants, data that was falsely recorded during those charging times was subsequently removed from the data. Further, RR and HR data were aggregated per second and single points in the data were deleted.

Only participants that had at least 24 h of ECG recording without much noise were included in the final analyses. This selection was performed based on visual inspection of the data by three independent researchers. Middle-aged participants were excluded from the current data analysis when there was no data collected (*N* = 6), when there was less than 24 h of recording (*N* = 17), or when more than half of the data was noise (*N* = 16). For the group that followed a slightly different protocol, only the data that was collected at home (starting at 00:00 h at the fourth study day until 12:00 h on the fifth day) could be included in the current analyses. However, of these 38 participants, 23 participants had to be excluded because no data was available on the fourth day (*N* = 18) or less than 24 h of data was available (*N* = 3), or more than half of the data was noise (*N* = 2). A total of 13 young participants was excluded from the current analyses after data cleaning due to having less than 24 h of recording (*N* = 7), more than half of the data was noise (*N* = 5), or no Equivital data was collected (*N* = 1). After data cleaning, we included a total of 73 middle-aged participants, comprising 37 offspring of long-lived families and 36 of their partners, and 35 young participants.

### Mean heart rate over 24 h and during sleep and awake periods

For the mean HR analyses, 24 h of data (starting at 00:00 h) with the most available data points per participant was selected. In principle, the second study day starting at 00:00 h until the third study day at 00:00 h was selected, but if the data from those 24 h was not of good quality, another 24 h (starting at 00:00 h) of data was selected. This selection was performed based on visual inspection of the data by three independent researchers. The mean of the selected 24-h data was calculated per participant. The 24-h data was split for sleep and awake periods using the individual bedtime and getting up time which was recorded by the participants in a dairy during the study period. The mean HR over the sleep and awake periods was calculated for each participant. A sensitivity analysis was performed excluding the middle-aged offspring and partners using medication known to influence HR (beta-blockers, antiarrhythmic agents, and beta-2-agonists).

### Heart rate variability

HRV analyses were performed using data of sleep and awake periods separately, split by using the individual self-reported bedtime and getting up time. HRV is defined as the variation in the time interval between successive beats. Here, Detrended Fluctuation Analysis (DFA) was used as a non-linear measure to assess HRV. DFA extracts the fluctuations between consecutive beat-to-beat intervals in non-stationary time series. Different numbers (bins) of consecutive RR values were assessed for their internal variability, leading to a number F, which is an indication for the HRV. For different sizes of the bins, these F-values were determined. Using linear regression techniques, the slope of the F function was determined for short-term correlations (alpha-1), indicating the barometer reflex, and long-term correlations (alpha-2) reflecting regulatory mechanisms [14, 37, 38]. The boundary between short-term correlations and long-term correlations was determined for each individual by investigating the F(n)/n-plot, where breakpoints clearly indicate the two separate regions [42]. For most individuals, the alpha-1 ranged from 4–40 beats, while alpha-2 ranged from 64-1000 beats, where a transition region was used to avoid errors.

An alpha approaching 0.5 is white noise, indicating a very flexible and reactive system that will be influenced too much by external perturbations, which is very noisy; an alpha approaching 1.5 is Brownian noise, indicating a very rigid and static system, which will not react to input from the outside world; an alpha approaching 1 is considered to be persistent long-range power-law correlations, representing the ideal mix between this flexibility and rigidity and therefore represents an optimally healthy system [37]. We used the RR intervals in the raw data without removed charging times. The DFA analyses were performed in MATLAB (version R2021a) using the method described in Gu et al., [43]. The alpha-1 and alpha-2 values were compared between the groups. Furthermore, a sensitivity analysis was performed excluding the middle-aged offspring and partners using medication known to influence HR (beta-blockers, antiarrhythmic agents, and beta-2-agonists).

### 24-h rhythms of heart rate

For the analyses on 24-h rhythms of HR, all available data of participants was used, meaning that the amount of data per participant ranged between 24 h to 90 h. Data was aggregated per 15 minutes for further analyses. To analyze 24-h rhythms of HR, linearized cosinor analyses with 24-h and 12-h harmonics [44] were performed on individual HR timeseries in R, version 3.6.2 [41]. For each participant, the model predictions were used to determine the mesor (midline of the 24-h rhythm), the peak and trough times, as well as the absolute amplitude (difference between the maximum and minimum value divided by two) and the relative amplitude (absolute amplitude divided by the mesor). The R^2^ was used to assess the goodness-of-fit of the cosinor analysis. A sensitivity analysis was performed excluding the middle-aged offspring and partners using medication known to influence HR (beta-blockers, antiarrhythmic agents, and beta-2-agonists). Additionally, we performed three types of sensitivity analyses to explore the robustness of our findings. The first sensitivity analysis investigated the effect of excluding participants with a poor cosinor fit. We repeated the cosinor analyses 1) excluding the participants with a r-squared of the fitted cosinor <10% and 2) excluding the participants with a r-squared <20% and repeated the linear regression analysis. The second sensitivity analysis that we performed explored whether the number of cycles influenced the cosinor results. We grouped participants based on their number of cycles (1 cycle = 24 h) and performed (circular) ANOVA analyses to compare the cosinor results between groups. The last sensitivity analysis that we performed investigated the effect of excluding participants with so-called double peaks (cosinor peaks during day and evening). Two independent researchers found consensus on which participants displayed double peaks. We then ran the cosinor analysis again with these participants excluded and compared the results to the original analysis.

### Statistical analysis

Descriptive statistics in SPSS version 25 (IBM SPSS Statistics, USA) were used to calculate the characteristics of the study participants. Data was presented as mean with standard deviation (SD) if normally distributed. For continuous variables, differences between groups were assessed by Independent-Samples *T* tests. For categorical variables, these differences were assessed using a Chi-squared test. Normality was assessed for all continuous variables by using visual inspection of the histogram and Q-Q plot and by performing the statistical normality test Kolmogorov-Smirnov. Non-normally distributed data was presented as median with interquartile range and differences in these variables between groups were assessed using nonparametric independent samples Mann-Whitney *U* tests.

Linear regression was used to compare variables for mean HR (mean HR over 24 h, and during sleep and awake periods), for HRV parameters (alpha-1 and alpha-2 values), and for 24-h rhythm of HR (mesor, amplitude, relative amplitude, minimum and maximum) between offspring and partners and between middle-aged and young participants. For all variables, assumptions for linear regression (normality, multicollinearity, and homoscedasticity) were checked using visual inspection of the histogram and Q-Q plot and by performing Kolmogorov-Smirnov tests, by using the variance inflation factor (VIF) values, and by visual inspection of the plots between the standardized predicted values and the residuals, respectively. All assumptions were met for all variables, expect for the absolute and relative amplitude measures, where a nonparametric independent samples Mann-Whitney *U* test was used without adjustment for confounders because of non-normality. Analyses were performed in SPSS version 25 (IBM SPSS Statistics, USA) and adjusted for calendar age and sex when comparing offspring and partners and for sex when comparing middle-aged and young. Getting up and bed times, were compared using circular ANOVA using the R package circular (version 0.4–93) [45] to account for the circular nature of these data. For the peak and trough times analyses, the assumption for equal kappa was met, but the von Mises assumption was not (all *p* < 0.01, Watson one-sample test). Therefore, the Watson-Wheeler test, which is a non-parametric alternative of the circular ANOVA, was used without adjustment for confounders (age and sex). For all statistical analysis, two-sided *p*-values below 0.05 were considered statistically significant.

## RESULTS

### Characteristics of study population

In the current analyses, a total of 37 middle-aged offspring (age range = 52–83 y) and 36 middle-aged partners (52–83 y) recruited from the LLS together with 35 young individuals (18–40 y) from the Switchbox Leiden Study were included after performing data cleaning. Characteristics of the studied individuals divided in subgroups are summarized in Table 1.

**Table 1:** Characteristics of the study population.

	**Offspring of long-lived parents (*N* = 37)**	**Partners of the offspring (*N* = 36)**	Middle-aged^*^ (*N* = 73)	**Young (*N* = 35)**	***P* value^+^**	***P* value^++^**
Female, *n* (%)	15 (40.5)	18 (50.0)	33 (45.2)	22 (62.9)	0.417	0.086
Age [years]	67.0 (6.18)	66.9 (6.23)	67.0 (6.16)	22.8 (3.96)	0.951	<0.001
**Age of parents^□^**					
Age of mother [years]	92.0 (9.50)^◊^	83.0 (13.0)^◊^	NA	NA	<0.001	NA
Age of father [years]	92.0 (21.5)^◊^	76.0 (14.0)^◊^	NA	NA	<0.001	NA
**Anthropometrics**					
Height [m]	1.73 (9.00)	1.72 (8.48)	1.73 (8.70)	1.74 (7.01)	0.611	0.291
Weight [kg]	76.7 (13.0)	76.4 (13.0)	76.6 (12.9)	69.6 (9.57)	0.921	0.005
BMI [kg/m^2^]	25.5 (3.44)	25.7 (3.25)	25.6 (3.33)	22.8 (2.38)	0.814	<0.001
Fat mass [kg]^‡^	22.7 (9.40)^◊^	23.9 (7.10)^◊^	23.0 (8.60)^◊^	14.6 (4.74)	0.596	<0.001
Waist-to-hip ratio^‡^	0.86 (0.13)^◊^	0.90 (0.07)^◊^	0.88 (0.07)	0.77 (0.06)	0.179	<0.001
**Cardiovascular risk**					
Current smoking, *n* (%)^^^	1 (2.70)	1 (2.80)	2 (2.70)	−	0.972	−
Alcohol >20 units per week, *n* (%)^‡^	3 (8.10)	3 (8.30)	6 (8.20)	0 (0.00)	0.971	0.165
Hypertension, *n* (%)^‡^	4 (10.8)	7 (19.4)	11 (15.1)	0 (0.00)	0.303	0.058
CVD, *n* (%)^‡^	4 (10.8)	6 (16.7)	10 (13.7)	0 (0.00)	0.467	0.073
Use of medication influencing HR, *n* (%)^×^	2 (5.4)	11 (30.6)	13 (17.8)	−	0.003	−
**Lipid levels**					
Total cholesterol [mmol/L]^‡^	5.81 (0.98)	5.51 (0.87)	5.66 (0.93)	4.58 (0.87)	0.172	<0.001
HDL cholesterol [mmol/L]^‡^	1.73 (0.59)	1.73 (0.52)	1.72 (0.55)	1.73 (0.43)	0.959	0.952
Triglycerides [mmol/L]^‡^	0.85 (0.46)^◊^	0.86 (0.59)^◊^	0.86 (0.55)^◊^	0.83 (0.25)	0.683	0.286
**Sleep**					
Getting up time on study day [h]	07:51 (00:13)	08:04 (00:11)	07:58 (00:12)	08:42 (00:21)	0.250	<0.001
Bedtime on study day [h]	23:30 (00:15)	23:50 (00:13)	23:40 (00:14)	00:17 (00:24)	0.112	0.009

Data presented as mean (standard deviation) unless otherwise stated. ^◊^Data presented as median (interquartile range). ^**□**^Age of parents indicate age at death or age at moment the questionnaire was taken. ^‡^Missing waist-to-hip ratio of one middle-aged partner and missing values of 13 young participants for fat mass, waist-to-hip ratio, alcohol, total cholesterol, HDL cholesterol, and triglycerides. Data of 14 young participants were missing for hypertension and CVD. ^^^Data of 21 young participants were missing, so this variable is excluded from the table. ^*^Data of offspring and partners combined. ^×^One offspring and four partners using betablocker, three partners using both betablocker and antiarrhythmic agent (dihydropyridine calcium channel blocker), one offspring and one partner using antiarrhythmic agent (dihydropyridine calcium channel blockers), one partner using beta-2-agonist (bronchodilator), and one partner using betablocker, antiarrhythmic agent (dihydropyridine calcium channel blocker), and thyroid hormone medication. No in detail medication data available for young participants. ^+^*P*-value between offspring and partners. ^++^*P*-value between middle-aged and young participants. Abbreviations: BMI: Body mass index; CVD: cardiovascular disease; NA: not applicable.

The age of parents differed significantly between offspring and partners (*P* < 0.001), which was expected since the offspring were selected based on having at least one long-living parent (and one long-living aunt or uncle) and the partners from the offspring serve as environmental and age-matched controls. Furthermore, the number of people using medication influencing HR is significantly (*P* = 0.003) higher in the partner group than in the offspring (11 (30.6%) vs. 2 (5.4%)). Medication known to influence HR included beta-blockers, antiarrhythmic agents (dihydropyridine calcium channel blockers), and beta-2-agonists. None of the participants were using any other medication that can influence HR, such as antidepressants, anxiety medication, non-dihydropyridine calcium channel blockers, desloratadine, theophylline or drugs (e.g. amphetamine or cocaine). Since the sample size is halved due to data cleaning process, we checked for differences in baseline characteristics between the full study population (*N* = 135) of the LLS participants in the Switchbox Leiden Study and the current study sample (*N* = 73). Levels of total cholesterol and triglycerides were significantly higher in the current study sample than in the full study population (data not shown) but no differences were observed between offspring and partners in any of the study samples.

When comparing all middle-aged participants (consisting of offspring and partners) to the young participants, all anthropometrics, except for height, differed significantly between groups (*P* < 0.01), with young participants having lower weight, lower BMI, lower fat mass, and lower waist-to-hip-ratio. Total cholesterol levels also differed significantly between young and middle-aged participants (*P* < 0.001), with the young participants having lower total cholesterol levels. However, levels of HDL cholesterol and triglycerides did not significantly differ between groups. Considering sleep, getting up and bedtimes differed significantly between the middle-aged and young group. Young individuals generally got up later (08:42 (00:21) h) than the middle-aged participants (07:58 (00:12) h) (*P* < 0.001). Furthermore, middle-aged participants went to bed earlier (23:40 (00:14) h) than young participants (00:17 (00:24) h) (*P* = 0.009) in general.

### Raw HR data plots over 3.5 days

**Figure 1:**
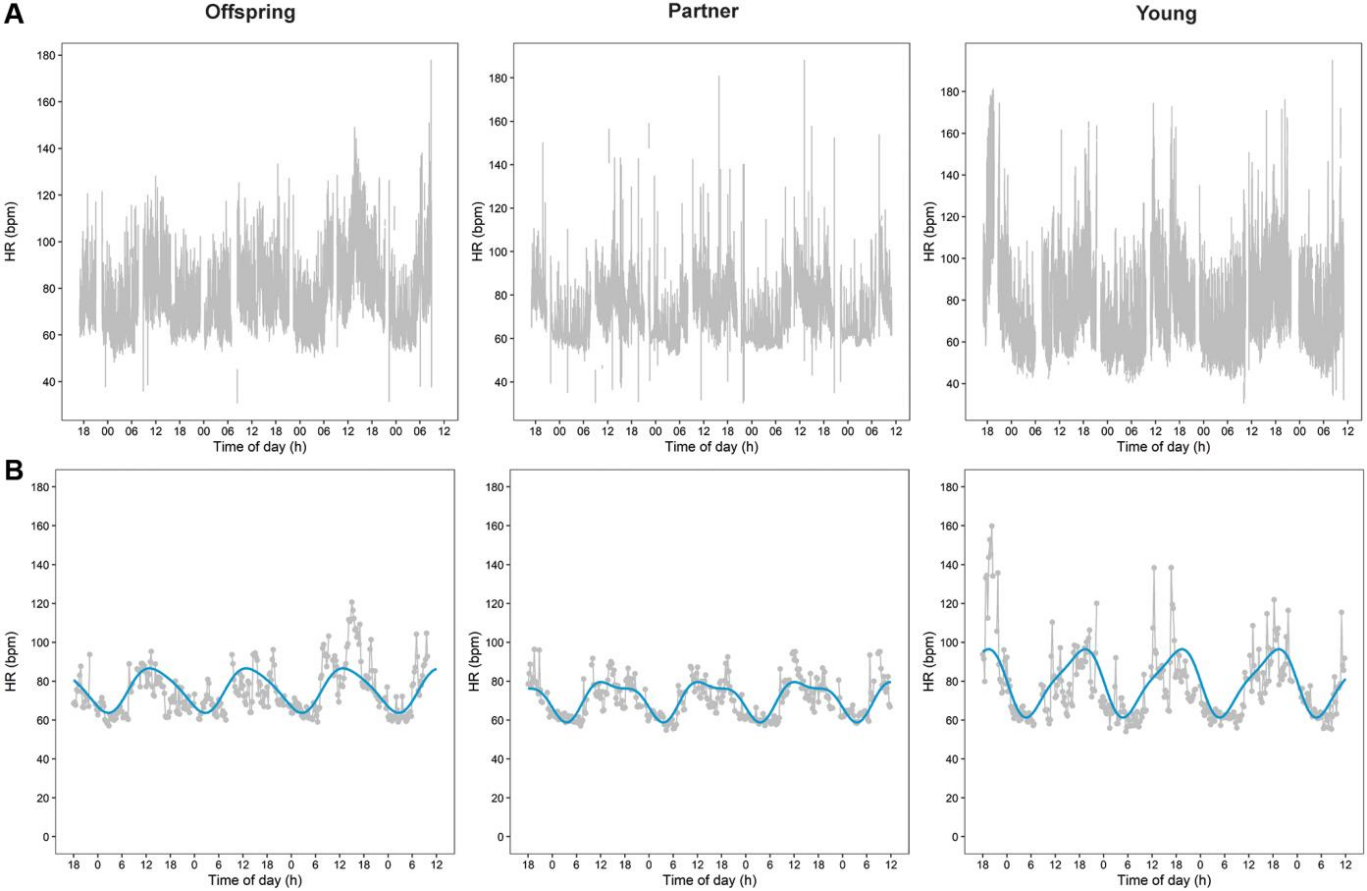
**Heart rate (HR) data plots over 3.5 days of three representative participants.** Plots of raw HR data during 3.5 days of three representative participants are presented in panel (**A**) One middle-aged offspring of a long-lived family, one middle-aged partner, and one young individual. Panel (**B**) Presents plots of the aggregated cleaned HR data of the raw data presented in panel A together with the cosinor plots resulted from the cosinor analyses.

In Figure 1A, we present plots of raw HR data during 3.5 days of three representative participants: one middle-aged offspring of a long-lived family, one middle-aged partner, and one young individual.

### Mean heart rate over 24 h and during sleep and awake periods

To assess whether HR is associated with familial longevity we measured the mean HR over 24 h and during sleep and awake periods in offspring and their partners. We found the mean (SD) HR over 24 h to be similar between offspring (71.49 (8.93) bpm) and partners (71.66 (7.45) bpm) (*P* = 0.929), also after stratification for sleep and awake periods (Table 2). When the analysis was repeated excluding the participants using medication known to influence HR (Supplementary Table 1), the difference in mean 24-h HR became larger between offspring and partners (71.91 (8.95) vs. 72.63 (6.74) bpm), but was still not significant (*P* = 0.734).

**Table 2:** Mean heart rate over 24 h and during sleep and awake periods in groups that differ in familial longevity status or chronological age.

	**Offspring of long-lived families (*N* = 37)**	**Partners of the offspring (*N* = 36)**	Middle-aged^*^ (*N* = 73)	**Young (*N* = 35)**	***P* value^+^**	***P* value^++^**
Heart rate - 24 h [bpm]	71.49 (8.93)	71.66 (7.45)	71.63 (8.18)	75.39 (8.80)	0.929	0.028
Heart rate - sleep period [bpm]	62.55 (7.28)	62.68 (7.24)	62.66 (7.21)	60.89 (6.26)	0.941	0.209
Heart rate - awake period [bpm]	77.09 (10.31)	76.75 (8.44)	76.97 (9.37)	83.42 (10.29)	0.874	0.001

Data presented as estimated mean (standard deviation). The linear regression analyses between offspring of long-lived families and their partners as controls were adjusted for sex and calendar age. The linear regression model analyses between the middle-aged group and young were adjusted for sex. ^*^Data of offspring and partners combined. ^**+**^*P*-value for difference between offspring of long-lived parents and their partners as controls. ^**++**^*P*-value for difference between middle-aged group and young.

Next we evaluated whether the mean HR over 24 h and during sleep and awake periods associates with age by comparing the middle-aged offspring and partners and the young participants. The mean (SD) HR over 24 h was significantly higher (*P* = 0.028) among the young (75.39 (8.80) bpm) than middle-aged participants (71.63 (8.18) bpm). When stratified for the awake period, the significant difference (*P* = 0.001) in mean (SD) HR even increased when young participants (83.42 (10.29) bpm) were compared to those of middle-age (76.97 (9.37) bpm). In contrast, the mean HR over the sleep period was not found to be significantly different. Most results remained comparable when excluding middle-aged participants using medication known to influence HR (Supplementary Table 1), but the difference in 24-h mean HR was no longer statistically significant (mean (SD) of 72.34 (8.06) vs. 75.36 (8.80), *P* = 0.087) between middle-aged and young participants.

### Heart rate variability

To study the relationship between heart rate variability (HRV) and familial longevity, we performed a DFA analysis during the sleep and awake periods and compared the results between offspring and their partners. All alphas found were very close to 1, indicating that all participants were healthy individuals. The alpha-1 (4–45) and alpha-2 (64–1000) did not significantly differ between offspring and partners (Table 3). When excluding offspring and partners using medication known to influence HR (Supplementary Table 2), the difference in alpha-2 became larger between offspring and partners, but not significantly (0.94 (0.12) vs. 1.01 (0.15), *P* = 0.055).

**Table 3:** Detrended fluctuation analysis (DFA) as a measure of heart rate variability (HRV) in groups that differ in familial longevity status or chronological age.

	**Offspring of long-lived families (*N* = 37)**	**Partners of the offspring (*N* = 36)**	Middle-aged^*^ (*N* = 73)	**Young (*N* = 35)**	***P* value^+^**	***P* value^++^**
**Sleep period**					
alpha-1 (4–45)	1.09 (0.19)	1.05 (0.22)	1.07 (0.20)	1.08 (0.12)	0.459	0.727
alpha-2 (64–1000)	0.95 (0.12)	0.98 (0.15)	0.96 (0.13)	0.85 (0.11)	0.285	<0.001
**Awake period**					
alpha-1 (4–45)	0.98 (0.15)	0.96 (0.18)	0.98 (0.17)	1.00 (0.13)	0.565	0.465
alpha-2 (64–1000)	1.09 (0.11)	1.11 (0.10)	1.10 (0.10)	1.04 (0.10)	0.331	0.002

Data presented as mean (standard deviation). The linear mixed model analyses between offspring of long-lived parents and their partners as controls were adjusted for sex and calendar age. The linear mixed model analyses between the middle-aged and young groups were adjusted for sex. Alpha-1 represents brief fluctuations and alpha-2 long-term fluctuations.^*^Data of offspring and partners combined. ^**+**^*P*-value for difference between offspring of long-lived parents and their partners as controls. ^**++**^*P*-value for difference between middle-aged group and young.

Next, to examine the relation between HRV and chronological age, we compared the results of the DFA analysis between middle-aged and young participants. Alpha-1 did not differ significantly between groups, but the alpha-2 difference was significant for both sleep (*P* < 0.001) and waking (*P* = 0.002) periods. In the sleep period, the average alpha-2 for young participants was 0.85, and for middle aged participants it was 0.96. In the waking period, the average alpha-2 for young participants was 1.04 and for middle aged participants it was 1.10 (Table 3). Sensitivity analyses excluding middle-aged offspring and partners using medication known to influence HR (Supplementary Table 2) did not materially change the results.

### 24-h rhythms of heart rate

The 24-h rhythms of HR were analyzed using cosinor analyses. Figure 1B presents plots of the aggregated cleaned HR data with cosinor plots during the same 3.5 days as the raw data presented in Figure 1A.

**Figure 2:**
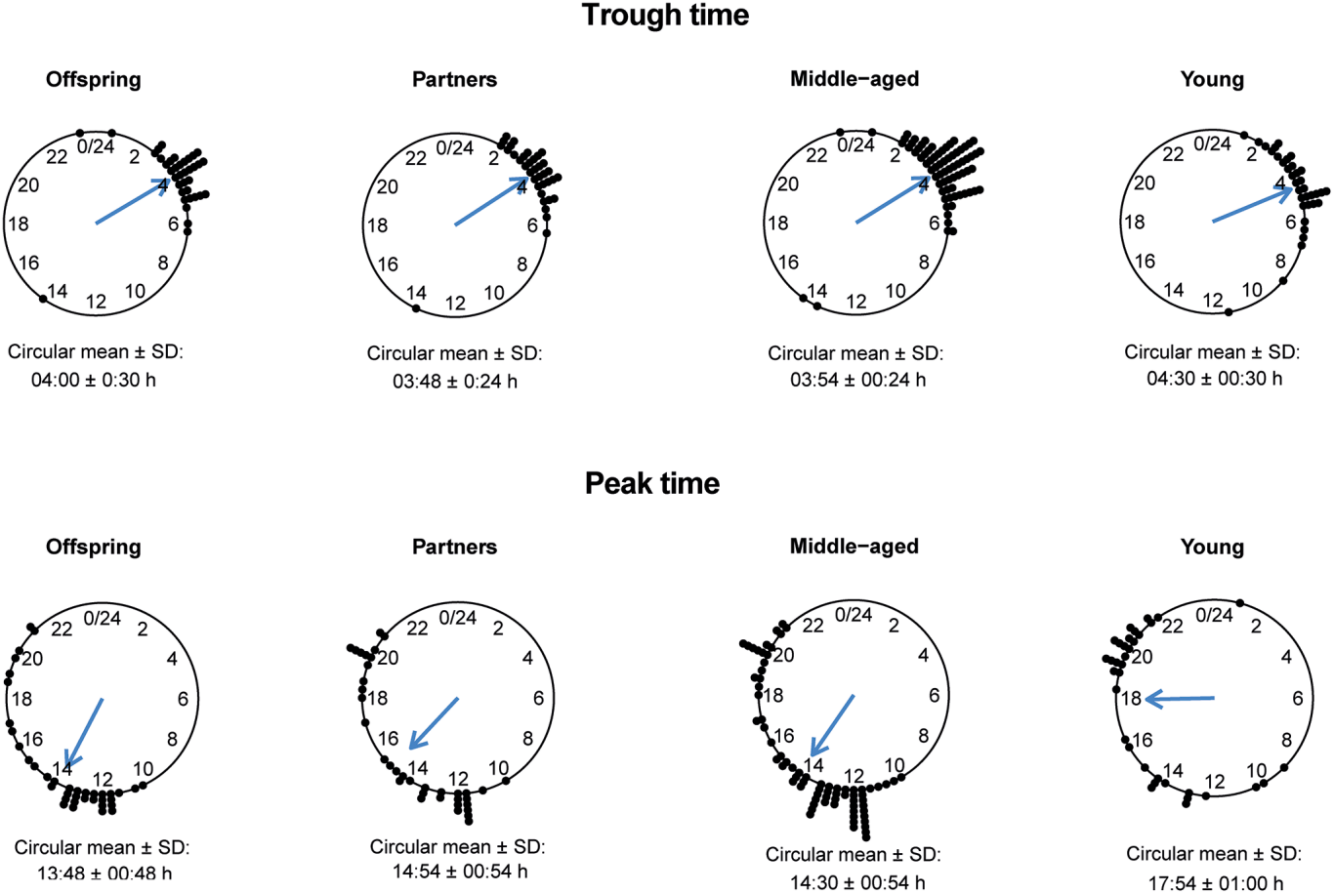
**Trough and peak times of 24-h heart rate (HR) data in offspring, partners, middle-aged and young participants.** The circular mean with standard deviation (SD) of the trough times (top panel) and peak times (bottom panel) are presented for groups of offspring, partners, middle-aged and young individuals.

To investigate the relationship between 24-h rhythms of HR over a period of 24 to 90 h and familial longevity, results of the cosinor analyses for offspring and their partners were compared. None of the parameters of 24-h rhythms of HR, such as the mesor, absolute amplitude, relative amplitude as a percentage, minimum, maximum, trough time, and peak time, significantly differed between offspring and partners (Table 4). In addition, the trough times for both offspring and partners had a narrow time window, while the peak times for both groups were more scattered around the clock (Figure 2).

**Table 4:** Measures of 24-h rhythms in heart rate in groups that differ in familial longevity status or chronological age.

	**Offspring of long-lived families (*N* = 37)**	**Partners of the offspring (*N* = 36)**	Middle-aged^*^ (*N* = 73)	**Young (*N* = 35)**	***P* value^+^**	***P* value^++^**
Mesor [bpm]	71.18 (7.55)	71.90 (7.82)	71.55 (7.65)	75.49 (7.51)	0.679	0.012
Absolute amplitude [bpm]^×^	10.99 (6.32)	10.76 (4.01)	10.93 (4.74)	14.82 (5.58)	0.886	<0.001
Relative amplitude percentage [%]^×^	14.90 (6.61)	15.63 (5.96)	15.07 (5.74)	19.43 (5.73)	0.982	<0.001
Trough time [hh:mm]^꙳^	04:00 (00:30)	03:48 (00:24)	03:54 (00:24)	04:30 (00:30)	0.501	0.027
Minimum heart rate [bpm]	59.31 (7.63)	60.34 (7.65)	59.79 (7.62)	58.64 (6.86)	0.560	0.448
Peak time [hh:mm]^꙳^	13:48 (00:48)	14:54 (00:54)	14:30 (00:54)	17:54 (01:00)	0.171	0.003
Maximum heart rate [bpm]	81.96 (9.70)	81.97 (9.93)	82.02 (9.75)	89.46 (10.04)	0.998	<0.001

Data presented as mean (standard deviation) unless otherwise stated. The linear mixed model analyses between offspring of long-lived parents and their partners as controls were adjusted for sex and calendar age. The linear mixed model analyses between the middle-aged and young groups were adjusted for sex. ^×^For these measures, data are presented as median (interquartile range) and non-parametric tests without correction for confounders were performed (Mann-Whitney U) due to no normal distribution. ^꙳^For these measures, data is shown as circular mean (standard deviation) and non-parametric circular tests without adjustment for confounders (Watson-Wheeler test). ^*^Data of offspring and partners combined. ^**+**^*P*-value for difference between offspring of long-lived parents and their partners as controls. ^**++**^*P*-value for difference between middle-aged group and young.

To test for association between 24-h rhythms of HR and chronological age, the results from the cosinor analysis were compared between the middle-aged and the young participants. The mesor, absolute amplitude, relative amplitude as a percentage, trough and peak times, and maximum HR differed statistically significant between the middle-aged and young participants (*P* < 0.05) (Table 4). The mean (SD) mesor in the middle-aged (71.55 (7.65) bpm) was lower than in the young participants (75.49 (7.51) bpm). In addition, the absolute amplitude as well as the relative amplitude were lower in the middle-aged participants than in the young participants. The mean (SD) maximum HR was also lower in the middle-aged group (82.02 (9.75) bpm) compared to the young group (89.46 (10.04) bpm). The trough times for the middle-aged group and the young group showed a very narrow time window, while the peak times for both groups showed a more scattered pattern (Figure 2). The trough and the peak times for middle-aged participants were significantly earlier in the day than for the young participants. However, the minimum HR did not differ significantly between the two groups.

Results did not materially change after excluding the middle-aged using medication known to influence HR, as presented in Supplementary Table 3. Sensitivity analyses were performed for all included participants to ensure the robustness of our findings. All three sensitivity analyses revealed no change in outcomes after excluding participants based on the sensitivity analyses (data not shown).

## DISCUSSION

In this study we examined the relationship between parameters of HR, HRV, and 24-h HR rhythms and familial longevity on the one hand, and chronological age on the other in data extracted from continuous ambulatory ECG measurements during 24 to 90 h. We did not find any association with familial longevity in the parameters of mean HR over 24 h or during periods of sleep and awake, nor in parameters of HRV, or in 24-h rhythms of HR since we did not observe any differences between healthy middle-aged offspring of long-lived parents and their partners. However, young participants showed a higher mean HR during 24 h and during the awake period, higher amplitudes, higher maximum HR, and later trough and peak times compared to the combined middle-aged participants. Furthermore, the HRV analysis using DFA showed a small, but significant difference between the middle-aged and young participants for long-term fluctuations in interbeat interval during both awake and sleep periods.

Associations between a low resting HR and longevity have been described in the literature [3], although we did not find any of such associations in our study. The middle-aged participants might be too young or too healthy to reveal such differences. Unlike what is observed for the LLS study as a whole (*N* = 1986), this subgroup of 73 offspring and partners from the Switchbox Leiden Study did not reveal significant differences in the prevalence of CVD endpoints or related risk factors. It is known that physical activity and lifestyle are important influencers of dynamics in HR. By design of the LLS, the offspring and partners are matched for adult environment which may explain the unexpected absence of a difference between offspring and partners in HR parameters. Associations of HR parameters to longevity in earlier studies may have been the result of beneficial environmental and behavioral factors rather than biological factors.

Studies showed that especially the maximal HR, measures of HRV, and the circadian rhythm in HR are changing with chronological age, in contrast to resting HR which does not seem to change [3, 11, 17, 26]. In our study, we indeed also did not observe differences between young and middle-aged participants in resting HR (during sleep period), except for an earlier trough time and a difference in HRV. Associations of HR parameters with chronological age were most prominent during the active period. The higher mean HR during the awake period (and consequently the higher HR amplitudes and maximum) and the later HR peak time in young compared to middle-aged participants could be the result of differences in exercise types, living situation (e.g. university/work vs. retirement) and day planning, including bedtime and getting up time (young individuals generally got up and went to bed later). Since participants continued with their daily lives during measurements, the intensity of daytime activity was not recorded or standardized. Therefore, we cannot discriminate whether the associations with chronological age are caused by environmental factors or by the biological aging process itself. The lack of standardization or control for activity is a limitation of the current study. Another limitation of this study is that no detailed information on medication use in the young participants was known and that older participants using medication known to influence HR were not excluded, although sensitivity analyses showed no major differences.

The DFA analysis showed that the alpha-2, which investigates long-term heartbeat variability, differed significantly between young and middle-aged participants. The alpha-2 values of our young participants were very similar to findings of Ivanov et al., for healthy individuals [46]. Pikkujämsä et al., looked at both alpha-1 and alpha-2 and showed that with aging, alpha-2 values increased [47]. In this study, young adults had an alpha-2 value of 0.99, middle-aged participants of 1.06, and elderly had a value of 1.12 [47], which is similar to the difference between young and middle-aged participants in our study. As the alpha-2 value is in between the middle-aged group and the elderly group in Pikkujämsä [47], our middle-aged participants seem to perform similar to this earlier report. However, we observed differences in alpha-2 between sleep and awake periods while Pikkujämsä et al., did not [47]. Healthy older adults from a study of Iyengar et al., had alpha-2 values further from 1 than our middle-aged participants, which can be explained by the older age of their study participants [48]. It is therefore well conceivable that the HRV of our middle-aged participants was still more ‘healthy’ than the HRV of the elderly in Iyengar et al., [48]. Schmitt and Ivanov found no difference between young and healthy older adults in alpha-2, but these recordings were for only two hours while sitting and watching a movie [49]. Our recordings were performed while participants continued with their daily and were for more than 24 h which may explain this difference. The higher alpha-2 value while waking in middle-aged participants compared to young participants indicates a less optimal reactivity and a heart that is less adaptable, which is in general less healthy. This finding might be a first indicator of an imbalance between sympathetic and parasympathetic nervous system activity associated with aging. Long-term fluctuations are implicated in complex mechanisms where the heartbeat is dictated by endocrine systems, metabolic processes, volume shifts, and other processes [48].

This is one of the first studies to look at the relationship between parameters of HR, HRV, and 24-h rhythms in HR based on continuous ambulatory ECG measurements over a period of several days with both familial longevity and chronological age in a single design. The difference in a HRV parameter for higher and long-term heart rates during both the sleep and awake periods in the middle-aged participants, compared to young, could be a first indication of deteriorated cardiovascular health in middle-aged individuals. This however small significant difference is particularly interesting since our middle-aged participants were relatively healthy. The significant associations between parameters of HR and daily rhythms in HR with chronological age specifically during the awake period could be due to behavior, health status or environmental factors besides the biological process of aging. Since we did not control for exercise or day planning in this study, the HR data in the sleep period might be most comparable between groups. In our study, we can conclude that resting HR during the sleep period is not associated with familial longevity or chronological age. This study showed that continuous ambulatory ECG measurements can be used to obtain adequate information on HR, HRV and 24-h rhythms in HR, which was also showed by others [50]. However, the small sample size, due to the poor quality of a part of the data, is a limitation of this study and should be improved in future studies. Furthermore, we suggest for future research to control for exercise and day planning between groups. Lastly we suggest to include an additional group with participants of an older age than the middle-aged group, and to investigate the relation between health status and HR parameters.

## Supplementary Materials

Supplementary Tables
