# Supplementary material for: The association between continuous ambulatory heart rate, heart rate variability, and 24-h rhythms of heart rate with familial longevity and aging
Source: Aging (Albany NY). 2022 Aug 16;14(18):7223–39. doi: 10.18632/aging.204219 (PMC9550250; doi:10.18632/aging.204219)
Supplement: Supplementary Tables [file aging-14-204219-s001.pdf]

SUPPLEMENTARY TABLES

Supplementary Table 1. Sensitivity analysis of mean heart rate over 24 h and during sleep and awake periods in groups that differ in familial longevity status or chronological age excluding participants using medication possibly influencing HR or HRV.

	Offspring of long-lived families (N = 35)	Partners of the offspring (N = 25)	Middle-aged* (N = 60)	Young (N = 35)	P value⁺	P value⁺⁺
Heart rate - 24 h [bpm]	71.91 (8.95)	72.63 (6.74)	72.34 (8.06)	75.36 (8.80)	0.734	0.087
Heart rate - sleep period [bpm]	62.73 (7.30)	63.10 (6.64)	62.98 (6.99)	60.87 (6.26)	0.843	0.139
Heart rate - awake period [bpm]	77.73 (10.27)	78.17 (7.21)	78.00 (9.07)	83.37 (10.29)	0.853	0.009

Data presented as estimated mean (standard deviation). The linear regression analyses between offspring of long-lived families and their partners as controls were adjusted for sex and calendar age. The linear regression model analyses between the middle-aged group and young were adjusted for sex. *Data of offspring and partners combined. ⁺P-value for difference between offspring of long-lived parents and their partners as controls. ⁺⁺P-value for difference between middle-aged group and young.

Supplementary Table 2. Sensitivity analysis of detrended fluctuation analysis (DFA) as a measure of heart rate variability (HRV) in groups that differ in familial longevity status or chronological age excluding participants using medication possibly influencing HR or HRV.

	Offspring of long-lived families (N = 35)	Partners of the offspring (N = 25)	Middle-aged* (N = 60)	Young (N = 35)	P value⁺	P value⁺⁺
**Sleep period**						
alpha-1 (4–45)	1.09 (0.19)	1.05 (0.24)	1.07 (0.21)	1.08 (0.12)	0.476	0.811
alpha-2 (64–1000)	0.94 (0.12)	1.01 (0.15)	0.97 (0.14)	0.85 (0.11)	0.055	<0.001
**Awake period**						
alpha-1 (4–45)	0.99 (0.15)	0.98 (0.19)	0.99 (0.17)	1.00 (0.13)	0.897	0.770
alpha-2 (64–1000)	1.09 (0.11)	1.12 (0.10)	1.11 (0.10)	1.04 (0.10)	0.343	0.003

Data presented as mean (standard deviation). The linear mixed model analyses between offspring of long-lived parents and their partners as controls were adjusted for sex and calendar age. The linear mixed model analyses between the middle-aged and young groups were adjusted for sex. Alpha-1 represents brief fluctuations and alpha-2 long-term fluctuations. *Data of offspring and partners combined. ⁺P-value for difference between offspring of long-lived parents and their partners as controls. ⁺⁺P-value for difference between middle-aged group and young.

Supplementary Table 3. Sensitivity analysis of measures of 24-h rhythms in heart rate in groups that differ in familial longevity status or chronological age excluding participants using medication possibly influencing HR or HRV.

	Offspring of long-lived families (*N* = 35)	Partners of the offspring (*N* = 25)	Middle-aged[*] (*N* = 60)	Young (*N* = 35)	*P* value[+]	*P* value[++]
Mesor [bpm]	71.65 (7.49)	72.88 (7.04)	72.21 (7.30)	75.47 (7.51)	0.514	0.039
Absolute amplitude [bpm][×]	11.44 (5.62)	11.90 (3.74)	11.44 (5.05)	14.82 (5.58)	0.605	0.001
Relative amplitude percentage [%][×]	15.07 (6.72)	16.10 (5.18)	15.56 (6.17)	19.43 (5.73)	0.524	<0.001
Trough time [hh:mm][☆]	03:54 (00:30)	03:42 (00:18)	03:54 (00:24)	04:30 (00:30)	0.491	0.030
Minimum heart rate [bpm]	59.59 (7.60)	60.29 (6.86)	59.82 (7.26)	58.64 (6.86)	0.711	0.439
Peak time [hh:mm][☆]	13:54 (00:48)	15:12 (00:54)	14:24 (00:48)	17:54 (01:00)	0.289	0.007
Maximum heart rate [bpm]	82.61 (9.56)	83.76 (9.42)	83.22 (9.45)	89.44 (10.04)	0.648	0.004

Data presented as mean (standard deviation) unless otherwise stated. The linear mixed model analyses between offspring of long-lived parents and their partners as controls were adjusted for sex and calendar age. The linear mixed model analyses between the middle-aged and young groups were adjusted for sex. [×]For these measures, data are presented as median (interquartile range) and non-parametric tests without correction for confounders were performed (Mann-Whitney U) due to no normal distribution. [☆]For these measures, data is shown as circular mean (standard deviation) and non-parametric circular tests without adjustment for confounders (Watson-Wheeler test) were performed. [*]Data of offspring and partners combined. [+]*P*-value for difference between offspring of long-lived parents and their partners as controls. [++]*P*-value for difference between middle-aged group and young.
